# Autonomy support in telehealth: an evolutionary concept analysis

**DOI:** 10.3389/fpubh.2025.1544840

**Published:** 2025-03-14

**Authors:** Yi Hou, Manyao Sun, Xueying Huang, Jiang Nan, Jing Gao, Nan Zhu, Yuyu Jiang

**Affiliations:** Department of Nursing, Wuxi School of Medicine, Jiangnan University, Wuxi, Jiangsu, China

**Keywords:** autonomy, autonomy support, concept analysis, patient autonomy, telehealth

## Abstract

**Aims:**

Autonomy support plays a critical role in safeguarding patients’ fundamental rights and promoting health behaviors. The context of autonomy support is transitioning from traditional face-to-face healthcare settings to telehealth, leading to an evolution in the connotation of autonomy support. This study aimed to clarify the connotation of autonomy support in telehealth and to develop a conceptual framework to guide innovations in clinical practice and the advancement of related theories.

**Methods:**

Rodgers’ evolutionary method was used to clarified attributes, antecedents, and consequences of autonomy support in telehealth. The integrative review strategy of Whittemore and Knafl was used as the methodology for searching relevant literature.

**Results:**

Twenty-five articles were included. The attributes were identified as: (i) technical feedback; (ii) virtual agent; (iii) choice; (iv) rationale; (v) empathy; (vi) collaboration; and (vii) strengths. The antecedents were: (i) telehealth service system; (ii) change in awareness toward autonomy support; and (iii) patient preference of needs for autonomy. The consequences were: (i) multidimensional perception; (ii) emotional experience; (iii) health behavior; (iv) social relation; and (v) technological dependence.

**Conclusion:**

This study clarified the attributes, antecedents, and consequences of autonomy support in telehealth, developing and improving a conceptual framework for autonomy support. These findings will provide a theoretical foundation for developing technology-enabled autonomy support strategies in telehealth practice, better adapting to the emerging needs of patients in the context of the digital age.

## 1 Introduction

Autonomy support is a key ethical principle in bioethics, as well as a fundamental aspect of nursing codes of ethics, reflecting respect for patients’ rights and values (1, 2). Self-Determination Theory (SDT) highlights that autonomy support increases patients’ motivation for self-care behaviors, sustains health-promoting actions, and improves health outcomes (3). In clinical practice, the primary agents of autonomy support are healthcare providers (HCPs), while the recipients are patients with health problems.

The concept of autonomy support has a long history and its connotation has evolved through several stages. Philosophers such as Socrates, Plato, and Aristotle have all explored autonomy in terms of human free will and moral responsibility (4). Kant elaborated on the support of individual autonomy, arguing that autonomy originates from free will (5). In healthcare, classical autonomy theory emphasized that autonomy support was about enabling patients to “truly” make decisions and choices in line with their values and desires, driven by the power of HCPs (6, 7). Autonomy support was first introduced as a terminology in SDT. From the perspective of positive psychology, SDT proposed that autonomy support is a social environment that fosters positive feelings, allowing individuals to experience a sense of freedom (8). According to this theory, the attributes of autonomy support included choice, rationale, and empathy (9). Building upon SDT, Kayser further developed the attributes of autonomy support to include choice, rationale, empathy, collaboration, and strengths. The attributes proposed by Deci and Kayser have served as theoretical guidance in the formulation of autonomy support interventions across different contexts, providing a direct pathway to understanding the connotation of this concept.

However, there is an inconsistency between the theoretical attributes of autonomy support and its practical application. In clinical practice, there is a lack of standardized guidelines for its implementation. Gillison, through a meta-analysis, outlined nine specific measures of autonomy support to guide clinical practice (3). Teixeira identified seven key strategies for autonomy support through expert consensus, providing a framework for the operationalization of autonomy support (10). In the literature, there are phenomena of “misuse” and “abuse” of the concept of autonomy support. For example, “autonomy-supportive consultation” emphasized the use of non-controlling language and respect for patient choice in face-to-face communication, highlighting partial attributes and the scenario of autonomy support (11). These show that the expression and application of this concept have become increasingly diversified, leading to confusion in clinical practice and systematic review.

As more and more scenarios of healthcare service delivery gradually transition to remote settings, these challenges may be further exacerbated. And in this setting, autonomy support is no longer only provided by HCPs through face-to-face communication, but more is automatically provided by HCPs preset procedures or agents according to the real-time environment (such as online website, automatic monitoring equipment, robots, etc.) (12–14). Moreover, due to the use of information and communication technology (ICT), the sources of health information provided by autonomy support are much broader and no longer solely dependent on the teaching of HCPs (15). Meanwhile, autonomy support in telehealth also brings a series of new problems for patients such as information overload, data protection, cybersecurity, etc. (16). Several studies have demonstrated that autonomy support strategies formulated based on Deci or Kayser’s attributes, when directly applied in remote settings, have significantly less positive impact on patients (17–19). Pettersson’s qualitative research also found that the characteristics of autonomy support have changed compared with previous studies (15). Rodgers proposed that concept is a dynamic process of evolution and transformation over time, influenced by various underlying factors including social environment, views and values, and life style (20). Consequently, it is necessary to clarify the attributes of autonomy support in telehealth in order to evade conflicts between care services and the diverse needs of patients, as well as the care service scenarios. This will reduce the potential adverse outcomes arising from a mismatch between supply and demand, and thereby significantly reducing the likelihood of these adverse implications of quality of life and health, while simultaneously supporting patient common rights of autonomy and dignity.

This study aimed to clarify the attributes, antecedents, and consequences of autonomy support in telehealth and to construct a conceptual model using Rodgers’ evolutionary approach, aiding HCPs in understanding its connotation in telehealth and guiding the design of telehealth services.

## 2 Methods

### 2.1 Concept analysis method

**Figure 1:**
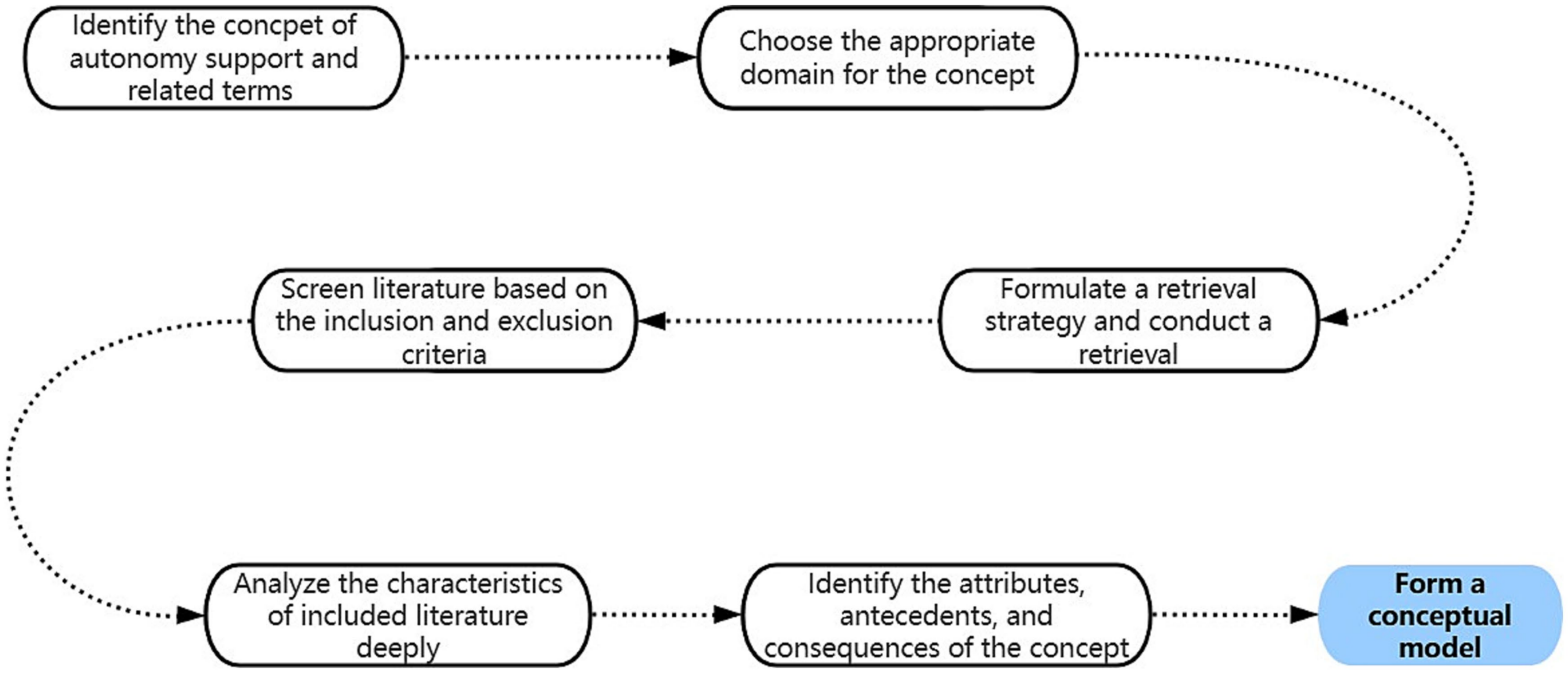
An overall flowchart.

Concept analysis is a systematic analysis process aimed at developing, clarifying, and refining a specific phenomenon. Rodgers’ evolutionary method and Walker and Avant’s classical method were the two most commonly used conceptual analysis methods (21). Walker and Avant’s method was applicable to the analysis of concepts that are relatively stable and well-defined. However, for dynamically changing or context-dependent concepts, this method may not be able to fully reveal their connotation (21). Rodgers’ approach emphasized that concepts can be affected by changes in practical application and essential characteristics over time (20). Rodgers emphasized the importance of identifying consensus by clarifying the status quo and the historical and evolutionary contexts of concepts, which involves six primary activities presented in Table 1 (22). These activities were concurrently conducted during the process of concept analysis, rather than being concrete steps in this process (20). Therefore, Rodgers’ evolutionary concept analysis approach was used as a methodology for analyzing autonomy support in telehealth, with a particular focus on the role of the background of the population in understanding this concept. An overall flowchart of this study was presented in Figure 1.

**Table 1:** Primary activities of Rodgers’ evolutionary concept analysis.

1. Specifying a concept and its alternate terminologies.
2. Determination and selection of the appropriate scope for data collection.
3. Collection of data related to: (i) the attributes of the concept; (ii) the contextual basis of the concept.
4. Data analysis based on concept attributes.
5. Identify an exemplar of the concept, if appropriate.
6. Determination of hypotheses or applications for further evolution of the concept.

### 2.2 Literature retrieval and data collection

#### 2.2.1 Data sources

##### 2.2.1.1 Dictionary

Chinese and English dictionaries were used to define the concept of autonomy support in telehealth. The English dictionaries include Oxford English Dictionary, Merriam-Webster Dictionary, and Collins Dictionary, while the Chinese dictionaries include Xinhua Dictionary and Cihai.

##### 2.2.1.2 Online database

Eight online databases were used to search the literature, including Pubmed, Embase, CINAHL Complete, PsycINFO, Web of Science, China National Knowledge Infrastructure, Wanfang and Sinomed.

#### 2.2.2 Definitions for autonomy support

The concept of autonomy support includes two sub-concepts of autonomy and support. Compared with other dictionaries that focus on macro-level explanations, for example, Oxford English Dictionary defines autonomy as “the condition or right of a state, institution, group, etc., to make its own laws or rules and administer its own affairs; self-government…” (23), Collins Dictionary has defined these two sub-concepts at the individual level. Therefore, in this study, the definition provided by Collins Dictionary was adopted. Collins Dictionary defines autonomy as “freedom to determine one’s own actions, behavior, etc.” (24), and defines support as “to give aid or courage to” (25). Autonomy support conveys “offer gid or encouragement for someone to take action, behavior, etc., on their own.”

#### 2.2.3 Search strategy

**Figure 2:**
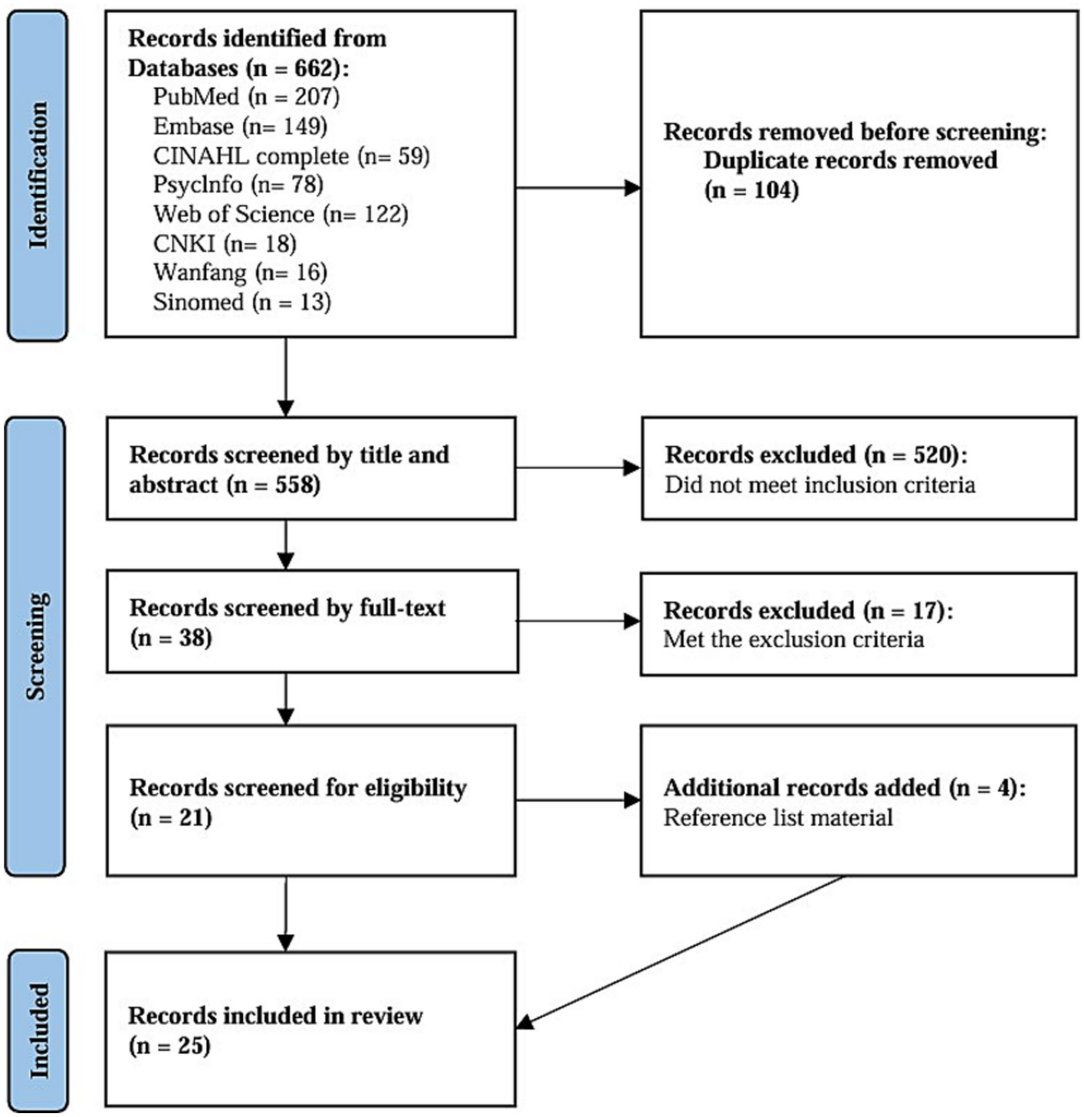
A PRISMA flow diagram.

The integrative review strategy of Whittemore and Knafl was used as the methodology for searching relevant literature in this study (26). This strategy included five steps, and its concrete process was as follows: (i) problem identification: identify the attributes, antecedents, and consequences of autonomy support in telehealth; (ii) literature search: the search keywords included “autonomy support” OR “autonomy promotion” OR “support for autonomy” OR “autonomy enhancing” OR “respect for autonomy” AND “telemedicine” OR “telehealth” OR “telenursing” OR “m-health” OR “e-health” OR “remote” OR “online” OR “portals” OR “website” OR “computer” OR “internet” OR “virtual”. The retrieval time was limited from the establishment of the database to 2024. The detailed literature search strategy can be found in Supplementary Table 1; (iii) data evaluation: screening of the literature was conducted based on the inclusion and exclusion criteria; (iv) data analysis: extract the attributes, antecedents and consequences from the included literature, and conduct repeated discussions and reviews; (v) presentation: A PRISMA flow diagram showing the search strategy process was presented in Figure 2.

#### 2.2.4 Inclusion and exclusion criteria

The inclusion criteria based on the Halfon (27) were: (i) the words “autonomy” and “support” in the title or abstract; (ii) providing a sufficient description of autonomy support; (iii) identifying an outcome related to autonomy; (iv) published in either Chinese or English.

The exclusion criteria were: (i) instant communication tools (e.g., telephone) was used as an adjunct after autonomy support such as during follow-up or data collection. However, within the implementation process of autonomy support, it was still the face-to-face interpersonal communication style; (ii) incorrect research types, such as published in non-peer-reviewed journals, editorials, and letters to the editor.

#### 2.2.5 Data collection

Two researchers first independently reviewed the title of the literature and determined that the basic content of the article was reflected in the provision of autonomy support in telehealth. The abstracts of the literature were then reviewed to determine that the literature met the inclusion criteria. For the literature that met the inclusion and exclusion criteria, two researchers read the full text and finally determined the results of the included literature. If there were any objections during this process, a third researcher will be invited to engage in discussion and make a collective decision regarding the inclusion of the literature. Three researchers repeatedly reviewed the included literature, held regular discussion meetings, and meticulously documented their perspectives during each session for synthesis, thereby establishing a shared comprehension of the concept of autonomy support in telehealth. After the preliminary identification of antecedents, attributes and consequences, relevant experts were invited to review again, and the results were confirmed as final when there were no objections. The discussion process was recorded by Tencent Meeting or live video, and EndNote 20 was used for literature management.

## 3 Results

**Figure 3:**
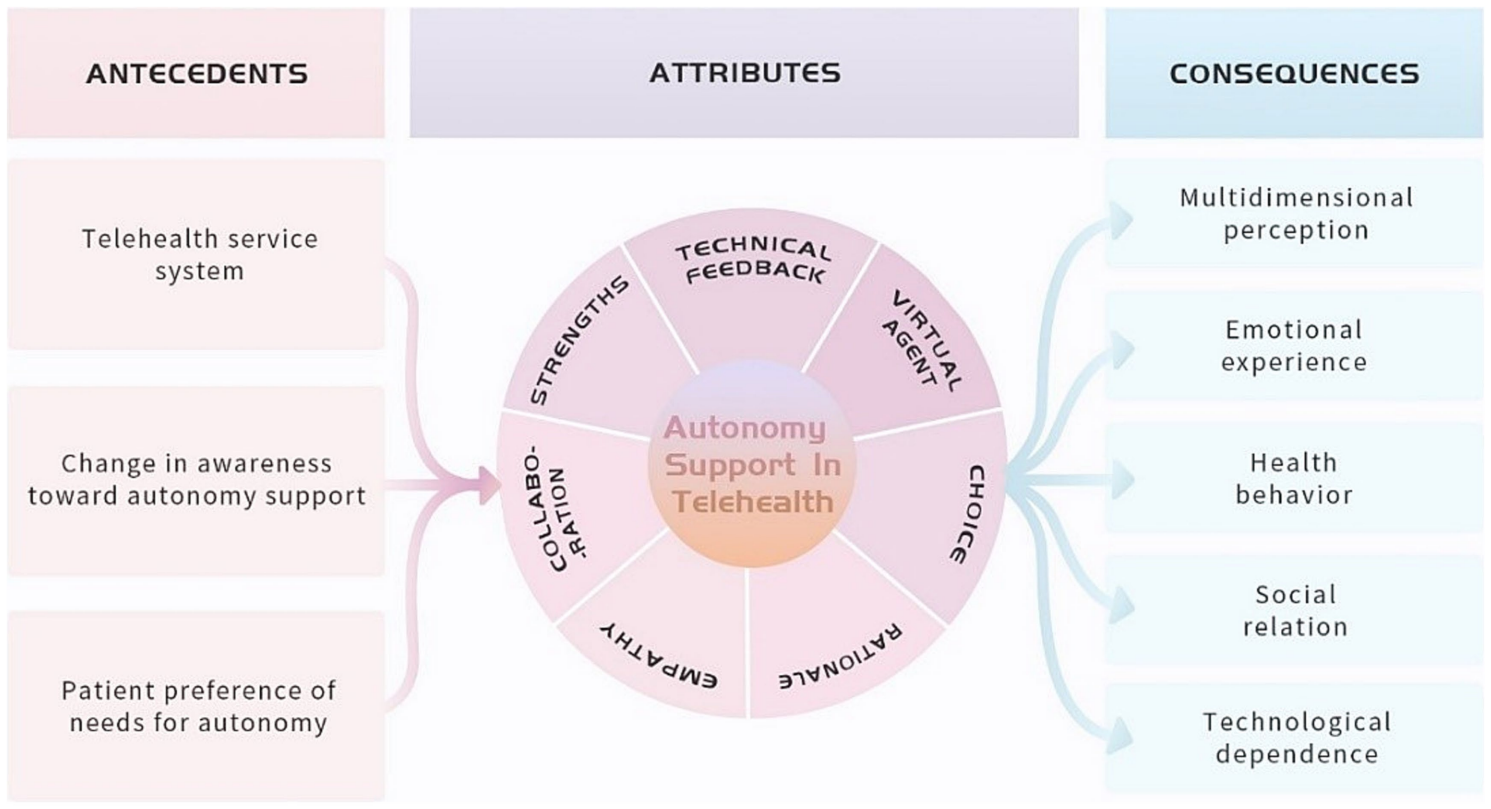
The conceptual model of autonomy support in telehealth.

25 studies were included in this concept analysis, and the detailed information was presented in Table 2. The conceptual model was presented in Figure 3. Furthermore, the sources and distribution of the attributes of autonomy support in telehealth were presented in Supplementary Table 2. The benefits and challenges brought by autonomy support in telehealth described in each study were presented in Supplementary Table 3.

**Table 2:** Main characteristics of included literature.

References	Study location	Article type	Discipline	Communication platform
Janssen and Schadenberg (35)	Netherlands	Conceptual framework	Multidisciplinary*	Robots*
Trzebiński et al. (14)	Germany	Quantitative	Multidisciplinary	Robots
Li et al. (13)	China	Quantitative	Nursing	Mixed media*
Kirkpatrick and Lawrie (12)	United States	Quantitative	Health communication	Mixed media
Kim et al. (29)	South Korea	Quantitative	Medicine	Mixed media
Cox et al. (31)	Australia	Mixed-methods	Nursing	Mixed media
van Strien-Knippenberg et al. (36)	Netherlands	Quantitative	Health communication	Mixed media
Legate and Weinstein (18)	United States	Quantitative	Multidisciplinary	Government-issued online news
Pettersson et al. (15)	Sweden	Qualitative	Psychology	Mixed media
Formosa (33)	Finland	Qualitative	Multidisciplinary	Robots
Altendorf et al. (17)	Netherlands	Quantitative	Medicine	Mixed media
Smit and Bol (41)	Netherlands	Quantitative	Multidisciplinary	Mixed media
Pirhonen et al. (34)	Australia	Expert opinion	Ethics	/
Gültzow et al. (32)	Netherlands	Protocol	Medicine	Mixed media
Bradshaw et al. (16)	Australia	Quantitative	Health communication	Government-issued online news
Altendorf et al. (19)	Netherlands	Quantitative	Nursing	Mixed media
Smit et al. (43)	Netherlands	Quantitative	Health communication	Mixed media
Lievense et al. (30)	Netherlands	Quantitative	Medicine	Mixed media
Altendorf et al. (37)	Netherlands	Quantitative	Health communication	Mixed media
Johnsen et al. (44)	Denmark	Quantitative	Nursing	Mixed media
Kim & Kim (39)	South Korea	Quantitative	Medicine	Mixed media
Kinnafick et al. (42)	United Kingdom	Quantitative	Nursing	Mixed media
Bång and Ragnemalm (38)	Sweden	Conceptual framework	Multidisciplinary	Mixed media
Resnicow et al. (45)	United States	Quantitative	Nursing	Mixed media
Williams et al. (40)	United States	Quantitative	Psychology	Mixed media

* Mixed media: formed by the integration of two or more digital media types, such as social media platforms, online conferencing, instant messaging tools, smart devices, and e-books. *Multidisciplinary: involving knowledge and methods from two or more disciplines. *Robots: capable of simulating human characteristics to start a real-time conversation and offer simple life assistance.

### 3.1 Attributes

The identification of defining attributes is the core of concept analysis. Defining attributes can distinguish the occurrence of a specific phenomenon from another similar or related phenomenon, thereby revealing the essential characteristics of a concept (28). The concrete content of each attribute was shown in Table 3.

**Table 3:** The concrete content of each attribute.

Attributes	Elements	Contents
Technical feedback	Assessment	Assess patients’ health status. Assess the barriers and problems the patients encounter. Assess patients’ concerns and potential difficulties. Assess patients’ personal characteristics (such as demographics, behaviors, preferences, attitudes, beliefs, self-efficacy, etc.). Assess the impact of the social environment on the patients. Guide patients in self-reporting their feelings and experiences (e.g., regarding received information, feedback from healthcare providers, use of technological devices, etc.).
	Analysis	Dynamic analysis of the patients’ health data, living environment, behavior patterns, health problems, and psychological condition.
	Supervision	Monitor the progress of patients’ goal and task completion, as well as changes in symptoms. Guide patients in completing the electronic diaries or health logs. Provide supervision from family members or professionals when necessary. Set up electronic monitoring, follow-up calls, and timely reminders.
Virtual agent	Intelligent interaction	Engage with the patients through online platforms or robots whenever possible. Provide digital devices with real-time chat functionality. Offer digital devices with web-based presence (telepresence) technology. Provide digital devices with emotional companionship features (e.g., companion robots).
Choice	Options and decisions	Ask patients if they are willing to make a choice. Provide options that are relevant and useful to the patients. Offer options prioritized in a clear order. Provide additional options beyond those directly related to the topic. Offer options within a reasonable range. Allow patients to choose their preferred mode of interaction with healthcare providers (e.g., video, phone, voicemail, etc.). Adjust options in real time based on patient preferences and context. Respect patients’ decisions in accordance with their own will and preferences.
	Opportunities	Provide patients with opportunities to implement their choices (e.g., achievable goals). Offer patients the opportunity to compare different options. Provide patients with opportunities to make reflective choices (e.g., allowing sufficient time for decision-making, responding promptly to patient questions, etc.). Give patients the opportunity to make choices free from environmental constraints (e.g., allowing patients to choose time, location, and equipment freely). Allow patients to make intuitive decisions and provide the opportunity to revise their choices.
Rationale	Customized information	Provide a brief introduction to the content and rules of the topic. Explain relevant knowledge and terminology. Clarify the rationale behind recommended activities, stating their importance, necessity, or urgency. Offer demonstrations or instructional content. Provide information that addresses patients’ actual needs and alleviates their concerns. Describe potential risks. Provide factual or evidence-based information. Offer widely accepted information aligned with values. Provide information about the best strategies. Share expert recommendations. Clarify the source of the information. Present social comparison results from similar situations. Indicate the healthcare provider’s stance on the information. Deliver information at the appropriate time. Push information that aligns with the patient’s current understanding. Push information that offers new insights for the patient. Provide cues to help patients make choices (e.g., listing important considerations).
Empathy		Understand and acknowledge the patients’ viewpoints, feelings, and opinions. Provide role-similar scenarios for the patient.
Collaboration	Remote communication	Actively inquire with the patients. Encourage the patients to ask questions and provide responses. Explore topics the patients wish to discuss. Facilitate peer-to-peer online communication.
	Goals and tasks	Express willingness to offer assistance. Invite the patients to participate in collaborative planning, setting goals and plans together. Clearly communicate tasks and goals. Develop tasks and objectives that align with the patients’ needs. Create realistic and achievable tasks and objectives. Formulate plans to address potential challenges. Assist the patients in setting their own goals, tasks, and plans. Establish long-term goals.
Strengths	Exploration	Guide the patients to actively explore problem-solving strategies. Express positive future possibilities to the patients. Encourage the patients to explore their motivation for self-change. Motivate the patients to try new approaches. Provide opportunities for the patients to experience success. Offer immediate rewards and praise for correct behaviors. Ensure the patients maintain hope (e.g., by postponing enjoyable events).
	Self-reflection	Encourage the patients to evaluate their own performance and behaviors. Encourage the patients to reflect on the outcomes of their behaviors, attribute success or failure, and engage in reflective thinking about the information received.

#### 3.1.1 Technical feedback

The process of autonomy support in telehealth is fundamentally reliant on technology-based feedback (29, 30). This feedback occurs across three stages: evaluation, analysis, and supervision (13). It is facilitated through remote monitoring technologies, data analysis tools, and telecommunication systems, which are automatically implemented through the inherent components and software of remote terminals (29). Both patients and HCPs can actively interact with these technologies to provide feedback, or rely on pre-programmed software for automatic feedback (29). The feedback includes real-time health status or electronic health records, personalized health recommendations, efficacy assessments, or experience reports, among other forms (30–32). In telehealth, technical feedback symbolizes professional oversight, boosting patient confidence and enhancing a sense of autonomy (31). Furthermore, autonomy support in telehealth provides timely reminders and electronic monitoring functions, which assist patients in completing self-management activities and further foster their autonomy (33, 34).

#### 3.1.2 Virtual agent

The characteristic of autonomy support in telehealth is the provision of virtual agent. Virtual agent is virtual entities equipped with intelligent interaction systems, which currently take the form of pre-programmed software or language models (14). For instance, telepresence robots can offer emotional support and strengthen patients’ connection to the outside world, while chatbots provide “light” companionship, and programs with telepresence systems create a sense of reality for patients (14, 34). Virtual agent not only provide practical health-related assistance but also establish a symbolic “expert” role (31). Virtual agent is designed to reduce patient loneliness and dependence on HCPs, enhance feelings of engagement, and compensate for emotional deficits, thereby indirectly maintaining patients’ sense of autonomy (34, 35). Additionally, virtual agent is considered non-judgmental, offering patients ample space to express themselves freely (35). When interacting with patients, virtual agent remains unbiased, refrain from value judgments, and avoid using offensive or demeaning language, thereby maintaining a non-judgmental stance.

#### 3.1.3 Choice

Choice encompasses options and decisions. Autonomy support in telehealth utilizes technologies such as databases and algorithms to quickly assess patients, filter through a vast array of options, and immediately present targeted options that are highly relevant to patients’ preferences and needs (32, 36). These options cover additional options that were overlooked by the patient and related to the target topic (19). Patients are able to make decisions according to their own desires. Virtual agent and technology not only protect patients’ choices from interference, but also help them make the right decisions (32). This includes providing opportunities for comparing options, ample time to reflect on choices, and the chance to reconsider decisions after an initial intuitive decision (13, 37). Autonomy support in telehealth directly offers patients meaningful options, enabling them to make quick and easy decisions, all while fostering a sense of freedom in both choice and decision-making throughout the process (38).

#### 3.1.4 Rationale

The characteristic of rationale in telehealth is customized information. Autonomy support provides patients with the reasons or principles for making choices and taking actions (39). The delivery of these principles or reasons relies on technological systems and devices, including digital communication technologies, mobile devices, system software, and a range of information collection, analysis, transmission, and presentation technologies (30). The principles or reasons provided by these systems are referred to as remote information. Autonomy support in telehealth filters vast amounts of information based on patients’ needs, beliefs, and values, and then pushes customized health information (36, 40). And two key factors are the information that is characterized by importance, necessity and urgency, as well as the information that addresses the patients’ concerns or barriers (16, 18). Furthermore, whether the presentation of information meets the patients’ preference for information is also an important factor of autonomy support in telehealth (41).

#### 3.1.5 Empathy

An important characteristic of autonomy support is the expression of empathy toward patients’ experiences and feelings. In telehealth, HCPs express empathy by acknowledging and understanding patients’ experiences, concerns and emotions; validating and affirming patients’ viewpoints and feelings; and offering comfort and encouragement by referencing social environment (14, 42). These expressions of empathy are conveyed indirectly through ways such as online responses, comments, and inquiries (36). Additionally, autonomy support in telehealth can provide patients with virtual role experiences, wherein virtual scenarios or stories are presented that resemble the patients’ own situations or experiences (30). This not only helps patients resonate with, understand, and identify with the virtual role, but also inspires and motivates them (31).

#### 3.1.6 Collaboration

One characteristic of autonomy support is the active communication and collaboration between HCPs and patients to address health problems, with power shared during the collaborative process. In telehealth, the focus of collaboration is goal- and task-oriented, aiming to jointly develop personalized health solutions and plans to address potential challenges (13, 43). The forms of collaboration include synchronous and asynchronous messaging, video conferences, remote video consultations, electronic health records, patient portals, online health education and guidance, online Q&A forums, smart voice assistants, chatbots, social media, and instant messaging applications (15, 42, 44). These technologies enable collaboration between HCPs and patients to transcend time and location constraints, while also facilitating patients’ ability to record and review the collaborative content, thereby enhancing the efficiency and continuity of the interaction. Furthermore, autonomy support in telehealth provides HCPs with the opportunity to establish a relationship of mutual respect and equality through transparent information sharing, shared decision-making, ongoing interaction, and personalized care (32, 36). This ensures that patients can actively participate in decision-making during their health management process, gaining more control and achieving power-sharing (13).

#### 3.1.7 Strengths

Autonomy support in telehealth encompasses four key advantages: active exploration, self-reflection, quantification and visualization. Patients are encouraged to actively explore and self-reflect in order to stimulate their inner potential to pursue a healthy life (13, 45). Guiding patients to explore problem-solving strategies and reflect on behavioral outcomes is the most important links (13). HCPs need to guide patients to actively try after exploration and reflection. Through continuous exploration, reflection and trial, patients have the opportunity to experience more successful experiences and build health confidence (29). In addition, patients’ ability to think and solve problems is enhanced during the process, further developing their autonomy to pursue proactive health (15). In telehealth, patients’ strengths are quantified and visualized, which positively influences their health behaviors, for instance, quantitative data such as the frequency of health activity completion and the level of change can be visually presented to patients in an intuitive and comprehensible manner through the use of graphical representations and dynamic videos (12).

### 3.2 Antecedents

Antecedents include events that should happen or exist before the concept (28).

#### 3.2.1 Telehealth service system

Telehealth service system refers to integrated systems that leverage modern communication, information, and internet technologies to provide telehealth services to patients (46). These systems typically consist of multiple modules, including teleconsultation, remote monitoring, online education, online collaboration, health data management, virtual group environments, and self-management tools (12, 44). As a “medium” for delivering autonomy support to patients, the purpose of the telehealth service system is to ensure the smooth delivery of autonomy support services in a convenient, efficient, and cost-effective manner (29). Additionally, the predesign of the telehealth service system’s functionalities is crucial. Key considerations include: ensuring patient information security, system compatibility, system complexity, system mobility, clear identification of stakeholders, usage instructions and common troubleshooting methods, as well as incorporating functionalities such as search capabilities, voice calling, and video communication (47).

#### 3.2.2 Change in awareness toward autonomy support

HCPs, as providers of autonomy support, often view autonomy support as synonymous with adhering to the principles of beneficence and non-interference with patients’ decisions (48). The remote healthcare environment, inherently characterized by less judgment and control, has significantly lowered this impact (35). However, this technology-driven remote environment has also introduced challenges such as information overload, technological biases, and data security problems. The focus of HCPs’ understanding of autonomy support has shifted from the traditional emphasis on respect and non-interference to patient-driven practices empowered by technology, such as actively providing feedback on healthcare experiences, seeking medical assistance, and engaging in real-time dynamic interactions (15). Furthermore, the diminished effectiveness of applying traditional autonomy support strategies in the remote environment has further accelerated this shift in HCPs’ awareness (17).

#### 3.2.3 Patient preference of needs for autonomy

Needs for autonomy refer to the desire of patients to exercise their inherent right to freedom, which is the inherent characteristic of patients (9). Different patients exhibit different preferences of needs for autonomy. Patients, with high levels of needs for autonomy, desire to choose their own ways to achieve goals. And patients, with low levels, rely on explicit recommendations from experts or peers (41). Whether the differences in preferences of needs for autonomy among population are considered is a key factor directly determining the effectiveness of autonomy support in telehealth (17). The factors, influencing patient preferences of needs for autonomy, include the extent of privacy concerns, personalized advertising, e-health literacy, age, gender, education, and intention to use electronic or smart devices (41).

### 3.3 Consequences

Consequences are events or things that take place as the outcome of a concept (28).

#### 3.3.1 Multidimensional perception

Patients exhibit diverse responses to autonomy support in telehealth that they receive, initially embodied in their perception, including perceived relevance, perceived information value, perceived threat to freedom, perceived capability, and perceived source credibility (31, 40, 43). Different levels of perception will affect the cognitive process of patients and further affect the experience of autonomy support in telehealth (17).

#### 3.3.2 Emotional experience

Patients inevitably reside in an integrated environment of physical society and virtual network that impedes them from achieving autonomy, and autonomy support in telehealth assists patients in overcoming these barriers (49). When this support can effectively overcome barriers, patients will experience autonomy and subsequently generate positive emotional experiences (15, 31). However, autonomy support in telehealth more or less fails to take into account all autonomy obstructing elements, leading to the possibility that patients may experience autonomy frustration, feelings of loss, loneliness and solitude, etc., resulting in negative emotional experiences (12, 31).

#### 3.3.3 Health behavior

Autonomy support in telehealth safeguards the integrality of patient psychological structure and creates comfortable external conditions (50). This will promote positive attitudes, wills, and intentions of healthy behavior, thereby converting into long-term healthy behaviors. Consequently, patients will produce greater expected outcomes and self-efficacy toward health-promoting behaviors; they will experience better health condition and quality of life; and they will develop a greater sense of trust and satisfaction with healthcare services (14, 34, 35).

#### 3.3.4 Social relation

Autonomy support in telehealth fosters a new symbiotic relationship between patients and technology, emphasizing the ideology of “patient-centered, technology-assisted.” Through telehealth, patients can engage in frequent communication with multiple HCPs, other patients, and family members who are not physically present (34). This efficient and intensive communication helps patients connect more closely with society (34). Additionally, patients can interact with technologies themselves, such as chatbots, health management platforms, and virtual assistants (14). These will increase social cohesion and social support, and create networks that are conducive to the smooth operation of society (16).

#### 3.3.5 Technological dependence

Autonomy support in telehealth fosters increasing reliance on technology by both HCPs and patients due to continuous use of technology. Patients gradually develop a reliance on digital tools to manage their health, which encourages proactive health management (13). HCPs, in turn, use digital tools to assist with certain tasks, thereby reducing their workload (15). This mutual use of technology enhances both the efficiency and quality of healthcare services. However, excessive dependence on technology may lead to the degradation of HCPs’ clinical skills and increased vulnerability to technical failures, potentially diminishing patients’ self-management capabilities and independent decision-making skills (32). Therefore, autonomy support in telehealth must ensure a balanced reliance on technology, avoiding overdependence that could negatively impact both patients and HCPs.

### 3.4 Related terms

#### 3.4.1 Autonomy support intervention

The term “autonomy support intervention” traditionally refers to interpersonal communication conducted in face-to-face settings (12). However, with the introduction of “autonomy support in telehealth,” the attributes of autonomy support intervention have evolved. Specifically, “technical feedback” and “virtual agent” have become distinctive attributes of autonomy support in telehealth. In this context, face-to-face interactions are no longer a prerequisite; instead, the use of digital technologies has become an indispensable component. It is important to note that many authors often use the term “autonomy support” combined with a specific setting or medium to describe autonomy support in telehealth, such as “autonomy support in short-form health videos” or “autonomy support in telerehabilitation” (12, 31).

#### 3.4.2 Respect for autonomy

“Respect for autonomy” and “autonomy support in telehealth” are both concepts centered around patient autonomy. However, “respect for autonomy” is a broader ethical principle commonly used in medical ethics, applicable to all healthcare settings, and emphasizes the freedom and dignity of patient decision-making (1). In contrast, “autonomy support in telehealth” is a context-specific practice that focuses on how digital technologies and non-face-to-face methods can help patients maintain a sense of autonomy during the process of receiving healthcare services.

## 4 Discussion

### 4.1 Findings

Compared to previous studies (particularly, five attributes that Kayser has clarified), this research identified two new attributes of autonomy support: “technical feedback” and “virtual agent” (51). These two attributes have been extracted from the systematic analysis of relevant research on autonomy support in telehealth conducted in recent years. The analysis was based on the elements of interveners, intervention strategies, intervention theories, intervention implementation processes and intervention quality control. And these have not been explored in other studies yet. Additionally, five previously clarified attributes—choice, rationale, empathy, collaboration, and strengths—were found to have changed in their connotation.

Technical feedback, as both a new and foundational attribute of autonomy support in telehealth, is integral to the entire process. The unique characteristics of technical feedback contribute significantly to autonomy support in telehealth, including: (i) immediacy; (ii) dynamism; (iii) continuity (12). These features of technical feedback not only enhance the efficiency and personalization of health management but also foster a stronger sense of patient engagement and control, further advancing their autonomy. Furthermore, technical feedback fundamentally differentiates autonomy support in telehealth from traditional forms of autonomy support. While traditional autonomy support relies on singular, often face-to-face, communication pathways and the periodic, passive delivery of feedback by HCPs, technical feedback emphasizes real-time, multi-modal information delivery (46). It is driven by active, data-driven responses rather than patient-initiated queries or HCP-directed interventions. The development of artificial intelligence (AI) makes it possible to automatically analyze patients’ “health portrait” through machine learning to provide predictive technical feedback in the future. In addition, AI possesses capabilities such as providing multilingual translation, facilitating cross-linguistic communication, automatically recording and summarizing conversations, and converting specialized terminology into layman’s language (52). These features contribute to enhancing autonomy support in cross-cultural contexts.

Virtual agent is a new attribute of autonomy support in telehealth. Patients may experience lower emotional connection and interaction in telehealth, which can weaken their trust in and reliance on healthcare services (53). Virtual agent helps mitigate this problem by simulating patient-provider interactions and providing a human-like interaction experience. For instance, robots may use greetings, empathetic understanding, and comforting language to engage with patients (54). This form of humanized relational support is a key factor in enhancing autonomy, as it makes patients feel respected and understood, thus encouraging greater participation in the healthcare process. However, excessive anthropomorphism of virtual agents can lead to the “uncanny valley” effect in some patients, where overly human-like behavior causes discomfort or even fear (14). Therefore, in the future, when utilizing AI to provide autonomy support, it is essential to explicitly clarify the tool-oriented nature of AI. This can be achieved through thoughtful design and guidance aimed at minimizing user discomfort. Key strategies include adhering to a human-centered approach, establishing robust oversight mechanisms, and defining clear usage boundaries, all of which contribute to enhancing the user experience while maintaining ethical and functional integrity.

This study also revised five original attributes of autonomy support in telehealth. Choice is no longer about providing a large number of options for patients to choose from, but rather about providing options that are filtered and relevant to the patient. The rationale is also no longer to provide a large amount of information, but rather information that is filtered to match the needs of the patient. This may be due to the precise screening and data-driven technology, so that patients do not have to face a large amount of irrelevant information, but can obtain the content that is most relevant to their own health status. Beyond traditional verbal empathy expressed by HCPs, virtual role experiences now enable patients to experience empathy in a risk-free environment. The application of advanced technologies like “digital twins” and “mixed reality” could further enhance the empathetic experience. Traditionally, collaboration focused on interpersonal communication and emotional expression, but in telehealth, the core of collaboration has shifted to information sharing and content exchange. With the support of technology, collaboration now emphasizes problem-solving and task progression rather than just interpersonal interaction (55). By visualizing and quantifying patients’ strengths, patients can more intuitively recognize their intrinsic health potential. This method is particularly effective in motivating patients from diverse backgrounds, helping them uncover their strengths and resources, which, in turn, enhances their engagement and autonomy in health management.

Moreover, when providing autonomy support in telehealth, it is crucial to address and mitigate the risks associated with various technological factors. To ensure data protection, it is essential to strengthen data privacy safeguards and enhance the transparency of data usage. To minimize data bias, the use of diverse datasets, alongside continuous monitoring and adjustment, is paramount. In order to bridge digital literacy gaps, simplifying user interfaces and offering comprehensive technical support are essential steps. Additionally, exploring cost-effective technological solutions can help reduce disparities in access to healthcare services. To address cybersecurity concerns, regular security audits, coupled with patient education and heightened awareness of online safety, are critical. Nevertheless, due to variations in communication barriers, comprehension abilities, values, beliefs, and social norms across different cultural contexts, as well as disparities in technological accessibility, educational resources, social support, and policy frameworks between regions, these factors can significantly influence the implementation of autonomy support. Therefore, when formulating and implementing autonomy support strategies, it is essential to thoroughly account for cultural and regional characteristics to ensure the adaptability and effectiveness of the services provided.

### 4.2 Autonomy support in telehealth and human-centered AI

The transformation of healthcare service systems is a crucial antecedent. Currently, autonomy support in telehealth primarily relies on mature internet-based communication technologies and computer systems. However, with the rapid advancement of AI, AI has become a core component within information communication networks (56). Specifically, deep learning, a branch of AI, possesses the ability to autonomously learn and optimize rules, offering significant potential for autonomy support in telehealth in the future. This AI-driven, human-like automated information communication technology does not entirely replace human labor but serves as a productivity tool utilized by humans. In this context, humans remain the central agents, with technology serving as the tool. Humanism emphasizes placing humans at the center, ensuring AI serves humanity, and promoting the development of human-centered AI (57). Therefore, from the perspective of human-centered AI, this study, in conjunction with the above conceptual framework, presents a roadmap (Table 4) for the development of autonomy support strategies in telehealth for future practice.

**Table 4:** A roadmap of autonomy support in telehealth.

Stages	Elements	Contents
The preparatory work	Telehealth service system	With the core needs and well-being of patients serving as the starting point and ultimate goal of the design, AI is integrated to profoundly enhance the overall patient experience.
	Patient preference of needs for autonomy	Leveraging big data analytics technology, in-depth exploration and precise identification of needs for autonomy are conducted.
The implementation process	Technical feedback	Utilizing AI, a comprehensive analysis of patients’ objective physiological data and subjective psychological experiences is conducted, providing more precise and personalized technical feedback.
	Virtual agent	Through AI, the abilities of virtual agents in emotion recognition and expression are augmented to better comprehend the emotional states of patients.
	Choice	AI possesses the capability to accurately identify patients’ values, preferences, and interests, and based on these, prioritize and recommend service choices to ensure patients receive those that best align with their individual needs.
	Rationale	Utilizing AI to recognize patients’ cultural backgrounds and subsequently provide cause explanations that are commensurate with their cognitive levels.
	Empathy	Leveraging AI, diverse and content-rich virtual character scenarios are constructed to provide an immersive service experience.
	Collaboration	Utilizing AI to continuously analyze patients’ behavioral patterns and working methods, service processes and interaction modes are gradually optimized, enhancing human-machine collaboration efficiency.
	Strengths	Utilizing AI for in-depth processing and analysis of patients’ health data to uncover potential disease progression patterns and health management trends.
Assessment and improvement		Utilizing AI to assess patients’ perceptions, emotional experiences, health behaviors, social relation, and technological dependence in a real-time and dynamic manner.

### 4.3 Limitations

There are some limitations. As telehealth gradually emerges as a new medical model and remains in the developmental stage, the number of existing studies was limited. Additionally, this study was limited to literature published in English and Chinese, potentially leading to the exclusion of relevant studies. Furthermore, this study primarily explored autonomy support from the field of healthcare, which may limit its applicability to other domains.

## 5 Conclusion

This concept analysis comprehensively clarified the attributes of autonomy support in telehealth, providing researchers, educators, and HCPs with a clear understanding and definition of its connotation and denotation. Additionally, it offered a framework and guidance for the development of autonomy support strategies, the innovation of related theories, as well as the research, application, and evaluation of autonomy support technologies in telehealth. This study can help to promote the healthcare service system to better adapt to the changes in medical service models under the current background of digital empowerment.
