# Corrigendum: Autonomy support in telehealth: an evolutionary concept analysis

**DOI:** 10.3389/fpubh.2025.1610264

**Published:** 2025-05-21

**Authors:** Yi Hou, Manyao Sun, Xueying Huang, Jiang Nan, Jing Gao, Nan Zhu, Yuyu Jiang

**Affiliations:** Department of Nursing, Wuxi School of Medicine, Jiangnan University, Wuxi, Jiangsu, China

**Keywords:** autonomy, autonomy support, concept analysis, patient autonomy, telehealth

In the published article, there was an error in the article type. Instead of “Review”, it should be “Conceptual Analysis”.

The authors apologize for this error and state that this does not change the scientific conclusions of the article in any way. The original article has been updated.

