# Supplementary material for: Autonomy support in telehealth: an evolutionary concept analysis
Source: Front Public Health. 2025 Mar 14;13:1544840. doi: 10.3389/fpubh.2025.1544840 (PMC11949869; doi:10.3389/fpubh.2025.1544840)
Supplement: Supplementary file 1 [file Data_Sheet_1.pdf]

Supplementary Material

1 Supplementary Table 1. Search strategies (taking PubMed as the example)

Search strategies:	
#1	“autonomy support*” [Title/Abstract] OR “autonomy promotion” [Title/Abstract] OR “autonomous support*” [Title/Abstract] OR “support for autonomy” [Title/Abstract] OR “support autonomy” [Title/Abstract] OR “respect for autonomy” [Title/Abstract] OR “respect autonomy” [Title/Abstract] OR “autonomy enhancing” [Title/Abstract] OR
#2	“Telemedicine” [MeSH Terms] OR “Mobile Health [Title/Abstract] OR “mHealth” [Title/Abstract] OR “Telehealth” [Title/Abstract] OR “eHealth” [Title/Abstract]
#3	“Remote Consultation” [MeSH Terms] OR “Consultation, Remote” [Title/Abstract] OR “Teleconsultation” [Title/Abstract] OR “Teleconsultations”[Title/Abstract]
#4	“Education, Distance” [MeSH Terms] OR “Distance Education” [Title/Abstract] OR “Distance Learning” [Title/Abstract] OR “Learning, Distance” [Title/Abstract] OR “Online Learning” [Title/Abstract] OR “Learning, Online” [Title/Abstract] OR “Online Education” [Title/Abstract] OR “Education, Online” [Title/Abstract] OR “Online Educations” [Title/Abstract] OR “Correspondence Courses” [Title/Abstract] OR “Correspondence Course” [Title/Abstract] OR “Course, Correspondence” [Title/Abstract]
#5	“Telenursing” [MeSH Terms]
#6	“Telephone” [MeSH Terms] OR “Telephones” [Title/Abstract] OR “Switchboard Service” [Title/Abstract] OR “Service, Switchboard” [Title/Abstract] OR “Services, Switchboard” [Title/Abstract] OR “Switchboard Services” [Title/Abstract]
#7	“Cell Phone” [MeSH Terms] OR “Phone, Cell” [Title/Abstract] OR “Phones, Cell” [Title/Abstract] OR “Cellular Phone” [Title/Abstract] OR “Cellular Phones” [Title/Abstract] OR “Phone, Cellular” [Title/Abstract] OR “Phones, Cellular” [Title/Abstract] OR “Telephone, Cellular” [Title/Abstract] OR “Cellular Telephone” [Title/Abstract] OR “Cellular Telephones” [Title/Abstract] OR “Telephones, Cellular” [Title/Abstract] OR “Cell Phones” [Title/Abstract] OR “Portable Cellular Phone” [Title/Abstract] OR “Cellular Phone, Portable” [Title/Abstract] OR “Cellular Phones, Portable” [Title/Abstract] OR “Portable Cellular Phones” [Title/Abstract] OR “Transportable Cellular Phone” [Title/Abstract] OR “Cellular Phone, Transportable” [Title/Abstract] OR “Cellular Phones, Transportable” [Title/Abstract] OR “Transportable Cellular Phones” [Title/Abstract] OR “Mobile Phone” [Title/Abstract] OR “Mobile Phones” [Title/Abstract] OR “Phone, Mobile” [Title/Abstract] OR “Phones, Mobile” [Title/Abstract] OR “Mobile Telephone” [Title/Abstract] OR “Mobile Telephones” [Title/Abstract] OR “Telephone, Mobile” [Title/Abstract] OR “Telephones, Mobile” [Title/Abstract] OR “Car Phone” [Title/Abstract] OR “Car Phones” [Title/Abstract] OR “Phone, Car” [Title/Abstract] OR “Phones, Car”
#8	“Patient Portals” [MeSH Terms] OR “Patient Web Portal” [Title/Abstract] OR “Portal, Patient Web” [Title/Abstract] OR “Portals, Patient Web” [Title/Abstract] OR “Web Portal, Patient” [Title/Abstract] OR “Web Portals, Patient” [Title/Abstract] OR “Patient Internet Portals” [Title/Abstract] OR “Patient Internet Portals” [Title/Abstract] OR “Patient Internet Portals” [Title/Abstract] OR “Patient Internet Portals” [Title/Abstract] OR “Internet Portal, Patient”

	[Title/Abstract] OR "Internet Portal, Patient" [Title/Abstract] OR "Internet Portals, Patient" [Title/Abstract] OR "Patient Internet Portal" [Title/Abstract] OR "Portal, Patient Internet" [Title/Abstract] OR "Portals, Patient Internet" [Title/Abstract] OR "Patient Web Portals" [Title/Abstract] OR "Patient Portal" [Title/Abstract] OR "Portal, Patient" [Title/Abstract]
#9	"Electronic Mail" [MeSH Terms] OR "Mail, Electronic" [Title/Abstract] OR "Email" [Title/Abstract] OR "Emails" [Title/Abstract] OR "E-Mail" [Title/Abstract] OR "E Mail" [Title/Abstract] OR "E-Mails" [Title/Abstract]
#10	"Television" [MeSH Terms] OR "Televisions" [Title/Abstract]
#11	"wechat" [Title/Abstract] OR "qq" [Title/Abstract] OR "QQ" [Title/Abstract] OR "YouTube" [Title/Abstract] OR "tiktok" [Title/Abstract]
#12	#2 OR #3 OR #4 OR #5 OR #6 OR #7 OR #8 OR #9 OR #10 OR #11
#13	#1 AND #12

2 Supplementary Table 2. The sources and distribution of the attributes of autonomy support in telehealth.

References	Attributes						
	Technical feedback	Virtual agent	Choice	Rationale	Empathy	Collabo-ration	Strengths
Janssen & Schadenberg, 2024(1)	√	√	√	√		√	
Trzebiński et al., 2023(2)	√	√	√	√	√		
Li et al., 2023(3)	√		√	√	√	√	√
Kirkpatrick & Lawrie, 2023(4)	√		√	√	√	√	
Kim et al., 2023(5)	√		√	√	√	√	√
Cox et al., 2023(6)	√		√	√		√	
van Strien-Knippenberg et al., 2022(7)	√		√	√		√	
Legate & Weinstein, 2022) (8)			√	√			
Pettersson et al., 2021(9)	√		√	√			√
Formosa, 2021(10)	√	√	√	√			
Altendorf et al., 2021(11)	√		√	√			

Smit & Bol, 2020(12)				√			
Pirhonen et al., 2020(13)	√	√	√	√			√
Gültzow et al., 2020(14)	√		√	√		√	
Bradshaw et al., 2020(15)	√		√	√			
Altendorf et al., 2020(16)	√		√	√		√	
Smit et al., 2019(17)	√		√	√		√	
Lievense et al., 2019(18)	√		√	√	√		
Altendorf et al., 2019(19)	√		√	√	√	√	
Johnsen et al., 2018(20)				√			√
Kim & Kim, 2017(21)	√		√	√	√	√	√
Kinnafick et al., 2016(22)	√		√	√		√	
Bång & Ragnemalm, 2012(23)			√	√			
Resnicow et al., 2008(24)			√	√	√	√	√
Williams et al., 2007(25)	√		√	√		√	√

3 Supplementary Table 3. The benefits and challenges brought by autonomy support in telehealth.

References	Benefits	Challenges
Janssen & Schadenberg, 2024(1)	Enhanced patients' sense of freedom and internal motivation for behavior change.	The norms and beliefs that guide the development of social robots will have an impact on the patient experience.
Trzebiński et al., 2023(2)	Enhanced the level of patients' perception of autonomy support and their intentions for behavioral	1. The expert endorsement failed to have a positive impact on the patient.<br>2. The emotions displayed by the

	change.	chatbot might undermine the patient's autonomy.
Li et al., 2023(3)	Improved the patients' self-management ability, anxiety and depression, and quality of life.	It is necessary to provide training and education on smartphone applications and related knowledge to patients.
Kirkpatrick & Lawrie, 2023(4)	Enhanced patients' receptivity to health information.	The use of self-supportive language (such as "can", "might", "probably") is not sufficient to change attitudes and intentions towards behavior.
Kim et al., 2023(5)	Enhanced the patients' perseverance.	The development of information technology equipment requires a high cost.
Cox et al., 2023(6)	Enhanced the level of patients' autonomy support.	After the intervention, the patient experienced a sense of loss.
van Strien-Knippenberg et al., 2022(7)	1. Enhanced the effectiveness of health information.<br>2. Reduced cost-effectiveness.	The intervention is highly effective for patients with high needs for autonomy, while it is only moderately effective for those with low needs.
Legate & Weinstein, 2022)(8)	Promoted the motivation for individual behavioral change.	Highlighting the significance and urgency of information content is more effective than traditional self-support strategies.
Pettersson et al., 2021(9)	Enhanced the sense of freedom, confidence and autonomy that patients experienced.	The characteristics of autonomy support have changed in telehealth.
Formosa, 2021(10)	Promoted the autonomy of patients.	Manipulation, deskillification and illicit surveillance will undermine patients' autonomy.
Altendorf et al., 2021(11)	Enhanced the level of patients' perceived autonomy support and reduce psychological resistance.	The autonomy support language did not have any impact on the patient.
Smit & Bol, 2020(12)	/	There are differences in the needs for individual autonomy.
Pirhonen et al.,	Reduced patients' reliance	The differences in cultures from various regions will affect the

2020(13)	on professionals.	autonomy support provided by robots.
Gültzow et al., 2020(14)	Iterated the tools providing autonomy support.	The effectiveness of the tool needs to be further verified.
Bradshaw et al., 2020(15)	Enhanced the security of information and the effectiveness of information transmission.	Autonomy support requires information security guarantee.
Altendorf et al., 2020(16)	Enhanced the level of patients' perceived autonomy support.	The autonomy support language did not have any impact on the patient.
Smit et al., 2019(17)	Enhanced self-efficacy and behavioral motivation.	/
Lievense et al., 2019(18)	Enhanced self-efficacy and experiential sense.	It is difficult to maintain the long-term effect of behavioral changes.
Altendorf et al., 2019(19)	Enhanced the intention for behavioral change.	The control information does not necessarily undermine the autonomy of patients.
Johnsen et al., 2018(20)	Promoted patients' self-care behaviors.	/
Kim & Kim, 2017(21)	Changed bad habits.	/
Kinnafick et al., 2016(22)	Enhanced the effectiveness of information and the cost of intervention	Autonomy support intervention needs to fully consider the frequency and timing of information push in relation to the suitability for individuals.
Bång & Ragnemalm, 2012(23)	/	/
Resnicow et al., 2008(24)	Enhanced patients' motivation for behavioral change and their satisfaction with the intervention.	Further exploration is needed to uncover the characteristics of individuals.
Williams et al., 2007(25)	Enhanced patients' self-management ability	/

4 References

1. Janssen S, Schadenberg BR. A Psychological Need-Fulfillment Perspective for Designing Social Robots that Support Well-Being. International Journal of Social Robotics. 2024:1-22.

2. Trzebiński W, Claessens T, Buhmann J, De Waele A, Hendrickx G, Van Damme P, et al. The Effects of Expressing Empathy/Autonomy Support Using a COVID-19 Vaccination Chatbot: Experimental Study in a Sample of Belgian Adults. JMIR Form Res. 2023;7:e41148.

3. Li Y, Yang W, Qu Y, Zhang X, Lin M, Fu L, et al. Effects of an autonomy-supportive intervention programme (ASIP) on self-care, quality of life, and mental health in heart failure patients: a randomized controlled trial. Qual Life Res. 2023;32(11):3039-52.

4. Kirkpatrick CE, Lawrie LL. Can Videos on TikTok Improve Pap Smear Attitudes and Intentions? Effects of Source and Autonomy Support in Short-Form Health Videos. Health Commun. 2023:1-13.

5. Kim S, Shin Y, Jeong Y, Na S, Han CE. Autonomy support encourages use of more-affected arm post-stroke. J Neuroeng Rehabil. 2023;20(1):116.

6. Cox NS, Lee JYT, McDonald CF, Mahal A, Alison JA, Wootton R, et al. Perceived Autonomy Support in Telerehabilitation by People With Chronic Respiratory Disease: A Mixed Methods Study. Chest. 2023;163(6):1410-24.

7. van Strien-Knippenberg IS, Altendorf MB, Hoving C, van Weert JCM, Smit ES. Message Frame-Tailoring in Digital Health Communication: Intervention Redesign and Usability Testing. JMIR Form Res. 2022;6(4):e33886.

8. Legate N, Weinstein N. Can We Communicate Autonomy Support and a Mandate? How Motivating Messages Relate to Motivation for Staying at Home across Time during the COVID-19 Pandemic. Health Commun. 2022;37(14):1842-9.

9. Pettersson B, Janols R, Wiklund M, Lundin-Olsson L, Sandlund M. Older Adults' Experiences of Behavior Change Support in a Digital Fall Prevention Exercise Program: Qualitative Study Framed by the Self-determination Theory. J Med Internet Res. 2021;23(7):e26235.

10. Formosa P. Robot autonomy vs. human autonomy: social robots, artificial intelligence (AI), and the nature of autonomy. Minds and Machines. 2021;31(4):595-616.

11. Altendorf MB, Smit ES, Azrout R, Hoving C, Weert J. A smoker's choice? Identifying the most autonomy-supportive message frame in an online computer-tailored smoking cessation intervention. Psychol Health. 2021;36(5):549-74.

12. Smit ES, Bol N. From self-reliers to expert-dependents: identifying classes based on health-related need for autonomy and need for external control among mobile users. Media Psychology. 2020;23(3):391-414.

13. Pirhonen J, Melkas H, Laitinen A, Pekkarinen S. Could robots strengthen the sense of autonomy of older people residing in assisted living facilities?—A future-oriented study. Ethics and Information Technology. 2020;22(2):151-62.

14. Gültzow T, Smit ES, Hudales R, Knapen V, Rademakers J, Dirksen CD, et al. An Autonomy-Supportive Online Decision Aid to Assist Smokers in Choosing Evidence-Based Cessation Assistance: Development Process and Protocol of a Randomized Controlled Trial. JMIR Res Protoc. 2020;9(12):e21772.

15. Bradshaw EL, Ryan RM, Noetel M, Saeri AK, Slattery P, Grundy E, et al. Information Safety Assurances Increase Intentions to Use COVID-19 Contact Tracing Applications, Regardless of Autonomy-Supportive or Controlling Message Framing. Front Psychol. 2020;11:591638.

16. Altendorf M, Hoving C, Van Weert JC, Smit ES. Effectiveness of Message Frame-Tailoring in a Web-Based Smoking Cessation Program: Randomized Controlled Trial. J Med Internet Res. 2020;22(4):e17251.

17. Smit ES, Zeidler C, Resnicow K, de Vries H. Identifying the Most Autonomy-Supportive Message Frame in Digital Health Communication: A 2x2 Between-Subjects Experiment. J Med Internet Res. 2019;21(10):e14074.

18. Lievense P, Vacaru VS, Liber J, Bonnet M, Sterkenburg PS. "Stop bullying now!" Investigating the effectiveness of a serious game for teachers in promoting autonomy-supporting strategies for disabled adults: A randomized controlled trial. Disabil Health J. 2019;12(2):310-7.

19. Altendorf MB, van Weert JCM, Hoving C, Smit ES. Should or could? Testing the use of autonomy-supportive language and the provision of choice in online computer-tailored alcohol reduction communication. Digit Health. 2019;5:2055207619832767.

20. Johnsen H, Blom KF, Lee A, Nørgaard B. Using eHealth to Increase Autonomy Supportive Care: A Multicenter Intervention Study in Antenatal Care. Comput Inform Nurs. 2018;36(2):77-83.

21. Kim HR, Kim HS. Autonomy-supportive, Web-based lifestyle modification for cardiometabolic risk in postmenopausal women: Randomized trial. Nurs Health Sci. 2017;19(4):509-17.

22. Kinnafick FE, Thøgersen-Ntoumani C, Duda J. The effect of need supportive text messages on motivation and physical activity behaviour. J Behav Med. 2016;39(4):574-86.

23. Bång M, Ragnemalm EL, editors. Persuasive Technology. Design for Health and Safety. Lecture Notes in Computer Science; 2012.

24. Resnicow K, Davis RE, Zhang G, Konkel J, Strecher VJ, Shaikh AR, et al. Tailoring a fruit and vegetable intervention on novel motivational constructs: results of a randomized study. Ann Behav Med. 2008;35(2):159-69.

25. Williams GC, Lynch M, Glasgow RE. Computer-assisted intervention improves patient-centered diabetes care by increasing autonomy support. Health Psychol. 2007;26(6):728-34.
